# Aging and Vascular Disease: A Multidisciplinary Overview

**DOI:** 10.3390/jcm12175512

**Published:** 2023-08-25

**Authors:** Jeanette A. Maier, Vicente Andrés, Sara Castiglioni, Alessandro Giudici, Emily S. Lau, János Nemcsik, Francesca Seta, Paola Zaninotto, Mariella Catalano, Naomi M. Hamburg

**Affiliations:** 1 Department of Biomedical and Clinical Sciences, Università di Milano, 20157 Milano, Italy; sara.castiglioni@unimi.it; 2 VAS-European Independent foundation in Angiology/Vascular Medicine, 20157 Milano, Italy; mariella.catalano@unimi.it (M.C.); nhamburg@bu.edu (N.M.H.); 3 Centro Nacional de Investigaciones Cardiovasculares (CNIC), 28029 Madrid, Spain; vandres@cnic.es; 4 Centro de Investigación Biomédica en Red de Enfermedades Cardiovasculares (CIBERCV), 28029 Madrid, Spain; 5 Department of Biomedical Engineering, CARIM School for Cardiovascular Diseases, Maastricht University, 6229 ER Maastricht, The Netherlands; a.giudici@maastrichtuniversity.nl; 6 GROW School for Oncology and Reproduction, Maastricht University, 6229 ER Maastricht, The Netherlands; 7 Division of Cardiology Massachusetts General Hospital, Boston, MA 02114, USA; elau6@mgh.harvard.edu; 8 Health Service of Zugló (ZESZ), Department of Family Medicine, Semmelweis University, Stáhly u. 7-9, 1085 Budapest, Hungary; janos.nemcsik@gmail.com; 9 Vascular Biology Section, Boston University Chobanian & Avedisian School of Medicine, Boston, MA 02118, USA; setaf@bu.edu; 10 UCL Research Department of Epidemiology & Public Health, University College London, London WC1E 6BT, UK; p.zaninotto@ucl.ac.uk; 11 Inter-University Research Center on Vascular Disease, Università di Milano, 20157 Milano, Italy

**Keywords:** aging, arteries, cardiovascular diseases

## Abstract

Vascular aging, i.e., the deterioration of the structure and function of the arteries over the life course, predicts cardiovascular events and mortality. Vascular degeneration can be recognized before becoming clinically symptomatic; therefore, its assessment allows the early identification of individuals at risk. This opens the possibility of minimizing disease progression. To review these issues, a search was completed using PubMed, MEDLINE, and Google Scholar from 2000 to date. As a network of clinicians and scientists involved in vascular medicine, we here describe the structural and functional age-dependent alterations of the arteries, the clinical tools for an early diagnosis of vascular aging, and the cellular and molecular events implicated. It emerges that more studies are necessary to identify the best strategy to quantify vascular aging, and to design proper physical activity programs, nutritional and pharmacological strategies, as well as social interventions to prevent, delay, and eventually revert the disease.

## 1. Introduction

Aging is an inexorable physiological process characterized by the gradual decline in cellular and tissue capabilities to adapt to stressors and to recover after damage, with consequent accumulation of senescent and dysfunctional cells, dysregulated metabolism, and impaired intercellular communication [1]. Aging is the primary risk factor for the development of cardiovascular disease (CVD), the leading cause of morbimortality worldwide [2,3]. Population aging and CVD prevalence have been increasing rapidly in recent decades in both the developed and developing world. For example, nineteen countries are projected to have at least 10% of their population aged more than 80 years by 2050, with many exhibiting CVD and other age-related disorders [4]. Accordingly, total direct medical costs associated with CVD in the United States and in the European Union are expected to triple between 2010 and 2030 [5,6]. With these grim statistics, a better understanding of the mechanisms by which aging predisposes to CVD is urgently needed to improve primary prevention and develop more effective therapies to ultimately promote healthy aging and reduce the vast medical and socioeconomic impact of age-related CVD. In this review, we focus on mechanisms of vascular aging including a summary of (1) structural and functional age-dependent alterations of arteries, (2) clinical tools for early diagnosis of vascular aging, and (3) potential underlying cellular and molecular mechanisms. To this end, we conducted a review of studies examining keywords “aging”, “vasculature”, “gender”, “stiffness”, “calcification”, and “biomarkers” published in English from 2000 to 2023 using PubMed, MEDLINE, and Google Scholar.

## 2. Vascular Aging

**Figure 1:**
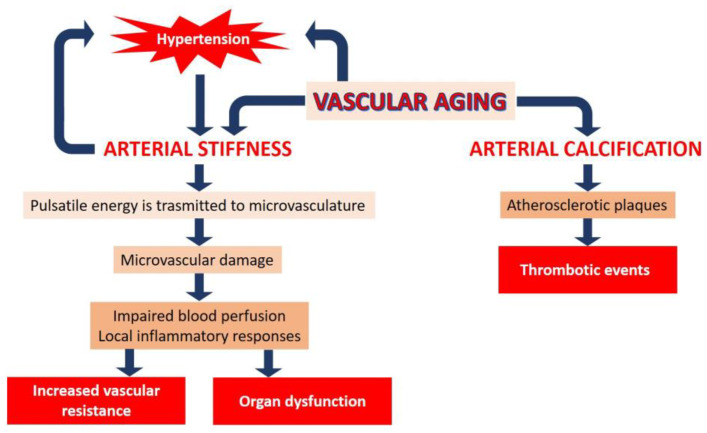
The effects of advancing age on the vasculature. Vascular aging is characterized by arterial stiffness and calcification. Arterial stiffness can induce an increase in vascular resistance and organ dysfunction. Arterial calcification induces thrombotic events. A feed-forward loop exists between vascular aging, hypertension, and arterial stiffness (see text for the details).

Vascular remodeling with advancing age is characterized by arterial stiffening and calcification (Figure 1). Arterial stiffening affects the macro- and micro-vasculature in unique ways. The main function of large arteries is to dampen the pulsatility of cardiac contraction by elastic recoiling after systolic expansion, so that constant, rather than pulsatile, blood flow is propelled to organs and tissues, including the coronary arteries. However, this buffering effect is lost as the aorta and its major branches stiffen in response to age, obesity, and other cardiometabolic diseases, and pulsatile energy is then transmitted to the delicate microvascular vessels of downstream organs and tissues. Microvascular damage can lead to impaired blood perfusion and local inflammatory responses, which can impair organ function and increase vascular resistance in the long term. The interdependence between hypertension and arterial stiffness, by which increased mean arterial pressure stimulates aortic wall maladaptive remodeling and further vascular stiffening, is particularly insidious as it establishes a detrimental feed-forward loop. The renal and cerebral vasculatures, which receive most of the cardiac output, are particularly sensitive to pressure pulsatility associated with arterial stiffness. Accordingly, a direct correlation between measures of arterial stiffness and cerebral and renal damage has been reported [7,8], underscoring the importance of targeted therapies to prevent arterial stiffness and, most importantly, associated target organ damage. 

An additional contributor to vessel wall stiffening is arterial calcification, which has been associated with adverse cardiovascular outcomes [9] and frailty in older adults [10]. The deposition of calcium phosphate occurs both in the media and in the intima, and may contribute to the necrotic core or along the shoulder of atherosclerotic plaques, which can induce thrombotic events and increase the risk of amputation [9]. Vascular calcification results from a complex, dynamic, and actively regulated process that, although not completely elucidated yet [11], involves the phenotypic reprogramming of vascular smooth muscle cells (VSMC) and altered cell–matrix interactions [12]. It is noteworthy that medial and intimal calcifications are independent diseases, but specific experimental models to understand the pathogenesis of medial calcifications are still lacking.

### 2.1. Biological vs. Chronological Aging: Lessons from Progeria

The extent of cardiovascular functional decline is highly variable among individuals of the same chronological age. Therefore, the identification of individuals for early prevention of CVD and other age-related disorders must rely on biomarkers of biological aging, also called physiological or functional aging, rather than chronologic age [13]. Because of the significant contribution of atherosclerosis and heart failure to CVD-related morbimortality in the elderly, substantial research efforts have been placed in understanding the genetic, molecular, and cellular mechanisms underlying CVD initiation, progression, and complications. Some hints derive from studies on Hutchinson–Gilford progeria syndrome (HGPS, OMIM 176670), an ultrarare (prevalence 1 in 18–20 million) genetic and fatal pediatric disorder characterized by segmental severe premature aging and early death and for which no cure exists [14]. The disease is caused by a de novo heterozygous dominant mutation in the *LMNA* gene (encoding nuclear A-type lamin) which causes the production of a truncated version of prelamin A called progerin. The accumulation of progerin causes multiple cellular alterations, including abnormal nuclear morphology, heterochromatin loss, mislocalization and loss of DNA damage repair proteins and chromatin-associated proteins, and mitochondrial and telomere dysfunction [15]. This results in accelerated VSMC senescence and loss, paralleled by reduced contractility, excessive deposition of extracellular matrix, and medial calcification [12,14]. In a murine model of progeria, vascular calcification is triggered by reduced extracellular deposition of pyrophosphate, a well-known inhibitor of vascular calcification [16]. Although patients with progeria lack or are only mildly affected by traditional cardiovascular risk factors, they develop CVD and die mainly from complications of atherosclerosis (myocardial infarction, heart failure, or stroke) at a mean age of 14.5 years [14]. HGPS research may therefore help identify mechanisms underlying CVD independently of risk factors or aging-associated chronic diseases that can influence cardiovascular health. Notably, progerin is expressed at low level in aged tissues of non-HGPS individuals, suggesting a role in normal aging [14]. Understanding how progerin causes CVD and premature aging may therefore shed some light on normal aging.

### 2.2. Sex Differences in Cardiovascular Aging

Sex differences in CVD are well established, and the mechanistic insights involved have been unraveled only recently [17]. While men and women have similar lifetime risks of developing CVD, women develop CVD later in life than men. Moreover, manifestations of CVD differ between men and women. For example, women with heart failure (HF) are more likely to have preserved left ventricular (LV) ejection fraction (EF) and non-ischemic etiology, while men often present with HF with reduced EF. Differences in cardiovascular aging between men and women may contribute to sex-based differences in CVD [18]. Early in life, men experience greater age-related vascular structural changes (intimal thickening, wall stiffening, calcium deposition, and atherosclerosis) and functional changes (endothelial dysfunction). However, after the sixth decade of life, age-related vascular dysfunction typically progresses at a faster rate in women than men. This observation has been consistently demonstrated in epidemiologic studies examining subclinical changes in vascular structure. The Baltimore Longitudinal Study of Aging measured carotid intima–media thickness (IMT)—a marker of early arterial wall alteration—over a 20-year period in 1067 men and women. The results showed that while men had higher baseline IMT than women, differences between men and women narrowed over time due to more pronounced acceleration of IMT in women later in life [19]. A similar pattern has been observed for other subclinical vascular measures, including coronary artery calcium scores [19]. 

Age-related decline in vascular function follows a similar trajectory to changes in vascular structure. Endothelial dysfunction decreases with age in men but is preserved in women until the onset of menopause, after which endothelial-dependent vasodilation markedly declines [20]. Arterial stiffness displays similar age-related trajectories in men and women, with lower autonomic tone, reduced baroreceptor response, and greater vascular function in pre-menopausal women vs. age-matched men. However, following menopause, women develop stiffer arteries than males. These differential patterns of vascular aging are reflected clinically in blood pressure (BP) trajectories over the life course. Data derived from the Framingham Heart Study enrolling 4993 individuals over a 28-year period show that early in life, male sex was positively associated with an increase in all BP measures, including systolic, diastolic, and mean BP, and pulse pressure, while the association with BP measures was attenuated in women. However, this association was attenuated in older individuals, as BP trajectories accelerated in women later in life [21]. 

Until recently, most investigations of sex differences in cardiovascular aging have operated under the prevailing hypothesis that cardiovascular aging is fundamentally the same in men and women, only delayed by 10–20 years in women. However, a recent investigation that examined BP trajectories over the life course in relation to sex-specific baseline values in 32,833 healthy individuals found that BP increases at a faster rate in women compared with men beginning early in life [22]. These results stand in contrast with the notion that cardiovascular changes in women lag behind men until the menopause transition, at which point cardiovascular aging accelerates preferentially in women. These data highlight the need to reimagine the design of studies examining sex differences in cardiovascular aging.

### 2.3. Assessment of Vascular Aging

**Figure 2:**
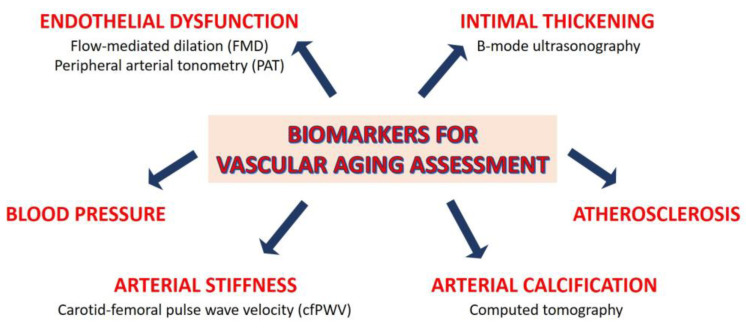
Biomarkers to assess vascular aging. Some invasive and non-invasive methods to measure vascular aging are reported.

Biomarkers of biological aging include a variety of molecular and cellular factors, such as telomere length, epigenetic alterations, somatic mutations, gut dysbiosis, inflammatory and omic-based biomarkers [23]. These factors can be integrated with functional and structural ones, such as arterial stiffness, blood pressure, endothelial dysfunction, intimal thickening, atherosclerosis, and arterial calcification. In the last decades, various invasive and non-invasive methods have been proposed to measure vascular aging (recently reviewed in [13,24]), some of which are summarized in Figure 2.

Endothelial dysfunction can be assessed by various non-invasive methods and is considered a good predictor of age-related vascular disease [25]. Flow-mediated dilation (FMD), which measures endothelium-dependent response to shear stress, is a well validated measure of endothelial dysfunction, which consistently declines over the lifespan until the age of 70 in men and age 80 in women. Sex differences in FMD correspond to age-related differences in coronary heart disease incidence and are present a decade before clinical CVD [20]. Remarkably, sex also affects the impact of some risk factors in (un)medicated individuals [26]. The endothelial function in microvascular blood vessels can also be noninvasively assessed using peripheral arterial tonometry (PAT) [27]. PAT quantifies the pulsatile volume change in the arteries at the fingertip, a phenomenon influenced in part by nitric oxide (NO) availability [28]. Despite the fact that PAT is reported to predict cardiovascular events and stroke [29], it is unsuitable for monitoring endothelial function in aging males [30]. The possibility of using serum biomarkers to evaluate endothelial function—soluble cell adhesion molecules, asymmetric dimethylarginine, glycocalix degradation products—is challenging [31]. There are few studies on these markers in the elderly population. While it is known that vascular endothelial glycocalyx is more vulnerable in older than in younger individuals [32], the circulating levels of glycocalyx breakdown products in the elderly remain poorly investigated [33].

The IMT, noninvasively and reproducibly measured through B-mode carotid ultrasound [34], is widely used to detect subclinical alterations in wall structure and to predict future overt cardiovascular events [35]. Cross-sectional studies show that IMT increases linearly with age [36]. Accordingly, individuals older than 65 years show higher IMT than younger people [37]. In both sexes, IMT increases with age and frailty [38].

Carotid–femoral pulse wave velocity (cfPWV) is the most accepted clinical marker of arterial stiffness, and is used for the detection of early vascular ageing [39]. While the stiffening of elastic arteries (e.g., the large arteries located in the proximity of the heart) is a normal process that characterizes chronological ageing, individuals with faster biological aging have accelerated progression of arterial stiffening, leading to increased cardiovascular risk compared with age-matched individuals. The SPARTE study has shown that the measurement of cfPWV allows for the enacting of strategies to slow down accelerated vascular ageing, potentially reverting the physiological trends [40]. A study calculating vascular age based on cfPWV was published on the Malmö Diet and Cancer Study Cohort [41]. However, an exact vascular age metric based on cfPWV is not freely available yet.

Another method to ascertain vascular age is via assessment of coronary artery calcium content (CAC) via computed tomography. CAC quantifies an individual’s risk of coronary heart disease. By comparing the individual’s CAC with age trends in the normal population, vascular age can be determined with an equivalent risk score. A simple conversion of CAC to vascular age can be achieved via the formula: vascular age = 39.1 + 7.25log(CAC + 1) [42]. 

As a single biomarker is often suboptimal to estimate biological age, a compounded scores which combines vascular imaging, functional tests, and physical, genetic, and biochemical parameters have been developed to improve CVD risk prediction (13). For example, the Framingham Risk Score (FRS) and the Systematic COronary Risk Evaluation (SCORE) calculate the CVD risk score, taking into account age (FRS and SCORE), total cholesterol (FRS and SCORE), HDL cholesterol (FRS), brachial systolic blood pressure (FRS and SCORE), sex (FRS and SCORE), smoking status (FRS and SCORE), and ongoing treatment of hypertension and diabetes (FRS) [43,44]. These scores provide a simple way to assess the difference between chronological age and vascular age. The vascular age can be interpreted as the chronologic age that carries the same estimated risk when all other risk factors are set to physiological values [44,45]. However, as composite predictors of biological age become more complex, their application in the general population becomes impractical and costly. In addition, a current limitation of different measurement-based or risk-score-based methods is that they do not provide equal estimations of the vascular age. As demonstrated in two recent papers [46,47], different methods may lead to different clinical decisions in preventive strategies. These observations suggest that studies aiming to define the “gold standard” method for vascular age calculation are compelling. Further studies are warranted to move vascular age calculation from bench to bedside [24].

## 3. Social Determinants in Vascular Aging 

In recent years, there has been growing interest in the social determinants of aging, which refers to the social, economic, and environmental factors that influence how people age. These factors can be both positive and negative, and they can impact the physical, mental, and social well-being of older adults. Some of the key social determinants of ageing include social isolation and loneliness, and socioeconomic status.

### 3.1. Social Isolation and Loneliness

Social isolation and loneliness are increasingly recognized as important public health issues affecting older adults. Social isolation refers to the objective lack of social connections or participation in social activities, while loneliness is the subjective feeling of being socially isolated or disconnected from others. Both social isolation and loneliness have been found to be associated with incident dementia [48] and poor cognitive function [49], and to unleash multiple vascular risk behaviors such as physical inactivity and smoking [50]. Loneliness, but not social isolation, was found to be associated with increased risk of chronic disease [51], including CVD [52]. Social isolation, but not loneliness, was found to be related to higher risk of mortality [53] and hospital admission for respiratory disease [54] and for falls [55]. Social isolation was also positively associated with increased blood pressure and C-reactive protein and fibrinogen levels [50].

A pivotal mechanism by which social isolation and loneliness influence health outcomes in the elderly is through their impact on mental health. Both social isolation and loneliness are correlated with higher rates of depression [56], which is considered a prevalent risk factor for CVD onset and associated mortality [57,58]. The link between depression and CVD is inflammation, which promotes endothelial dysfunction, a condition detected in depression [58]. Consequently, specific inflammatory cytokines or pathways might represent potential targets for the prevention and treatment of these concurrent diseases.

### 3.2. Socioeconomic Status

Socioeconomic status (SES) is another critical social determinant of ageing. Individuals with lower SES experience worse health outcomes compared with those with higher SES [59,60,61,62]. Notably, older men and women in the poorest SES groups can expect to live eight to nine fewer years free from disability compared with people in the richest SES groups [63]. Furthermore, low SES has been shown to be related to accelerated aging across a broad range of functional abilities and phenotypes independently of the presence of health conditions [64].

SES can influence health outcomes in older ages, mainly through lifestyle factors. Individuals with lower SES are more likely to engage in unhealthy behaviors such as smoking, excessive alcohol consumption, and physical inactivity. These behaviors are associated with higher rates of chronic disease and mortality at older ages. Moreover, SES affects access to healthcare services. Individuals with lower SES are less likely to have health insurance, less likely to access preventive care, and more likely to delay care when they are sick. This can lead to poorer health outcomes and higher rates of chronic disease and disability.

## 4. Cellular and Molecular Mechanisms Involved in Vascular Aging

At the molecular and cellular levels, the complexity of aging in mammals has been recapitulated in twelve intertwined hallmarks, among which mitochondrial dysfunction, oxidative stress, chronic low-grade inflammation, telomere attrition, epigenetic alterations, deregulated nutrient sensing, and cellular senescence [23] are all relevant also in driving vascular aging.

(1) Mitochondrial dysfunction: Impaired mitochondrial function in vascular cells reduces adenosine triphosphate (ATP) generation and increases reactive oxygen species (ROS), events that undermine the critical role of these cells in maintaining the integrity of the blood vessels.
(2) Oxidative stress: The generation of ROS, beyond the cellular anti-oxidant capacity, leads to inactivation of NO, a key regulator of vascular homeostasis. NO relaxes VSMCs, inhibits their proliferation, and exerts anti-thrombogenic actions [65]. In addition, aberrant ROS generation can impair the function of proteins, lipids, and DNA by inducing oxidative post-translational modifications, negatively affecting vascular homeostasis [66].
(3) Chronic low-grade inflammation: ROS activate nuclear factor (NF) kB, a transcription factor that orchestrates inflammation, resulting in endothelial release of cytokines, chemokines, and other inflammatory mediators. Indeed, age-related activation of inflammatory processes plays a key role in various macro- and micro-vascular disorders. Inflammatory cytokines re-shape endothelial function and promote senescence [67,68]. Moreover, endothelial cells (ECs) are one of the first cell types to become senescent with advancing age [67], promoting dysfunction and senescence of neighboring vascular cells, including VSMCs. Senescent ECs secrete transforming growth factor (TGF) β, which stimulates the synthesis of collagen and matrix metalloproteases [68], contributing to pathological remodeling of the vascular wall. Similarly, senescent VSMCs release pro-inflammatory factors such as IL6, leading to the activation of the IL6/STAT3 pathway which, in the setting of oxidative stress, stimulates the switch of VMSCs from a contractile to a synthetic phenotype [69,70] and consequent extracellular deposition of calcium phosphate. It has been estimated that VSMC-related mechanisms contribute ~50% to aortic wall stiffness with aging via an increase in the material stiffness of the aortic wall and/or vaso-actively regulating the aortic diameter.
(4) Telomere attrition and epigenetic alterations: All somatic cells have a limited lifespan because of the shortening of telomeres, an event which impairs end replication [23]. Of interest, short telomeres in vascular cells within the atherosclerotic plaque have been linked to a higher risk of CVD [71]. It is also emerging that telomere uncapping, i.e., the breakdown of their loop structure, is a better marker than telomere length in defining vascular aging [72]. Moreover, mounting evidence supports the role of epigenetics in vascular aging. Modifications of DNA and histones as well as non-coding RNA result in aberrant transcription and, therefore, in vascular cell dysfunction, vascular homeostatic imbalance and pathological remodeling [73,74].
(5) Deregulated nutrient sensing: Diet has a significant impact on aging [75], and aging impairs the cellular pathways implicated in energy sensing [76], including mammalian target of rapamycin (mTOR), AMP-activated protein kinase (AMPK), and sirtuins (SIRT), which control cellular behavior in response to nutrient availability, thereby influencing cell fate [76]. The mTOR inhibitor rapamycin reverses age-associated arterial dysfunction and decreases vascular stiffness and oxidative stress [77]. Similarly, AMPK confers vasoprotective effects by ameliorating endothelial function and inhibiting nuclear factor kappa-light-chain-enhancer of activated B cells (NFkB) and, consequently, inflammation [78]. SIRT and, in particular, SIRT1 exerts beneficial effects on the vasculature through its anti-inflammatory and anti-oxidant actions [79], decreasing obesity-induced vascular stiffness.
(6) Mechanical stress is an additional contributor to vascular aging. Unlike collagen, which can be actively synthesized over a lifetime, elastin, the fundamental component of large artery elastic compliance, is synthesized only during embryonic development and it has a very long half-life (~40 years) [80]. However, the cyclic strain from cardiac contraction deteriorates elastin lamellae over time. Moreover, calcium deposits in the media promote the destruction of elastic fibers. Collagen fibers are then deposited and/or structurally rearranged in response to fragmented elastin, leading to intrinsically stiffer elastic fibers. Decline in NO bioactivity with age further contributes to aortic wall stiffening via excessive extracellular matrix (ECM) protein crosslinking, in part by the activation of transglutaminase-2 and other enzymes [81].

**Figure 3:**
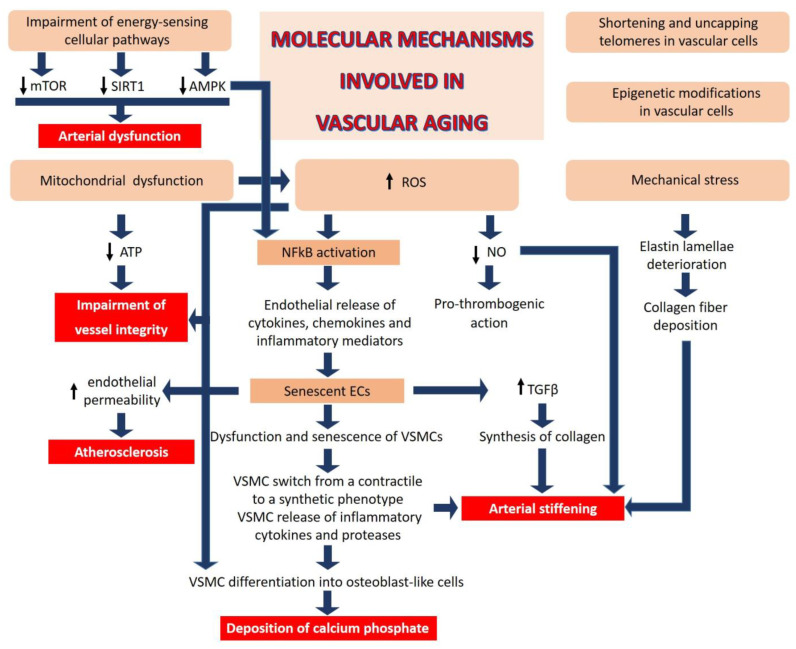
Molecular mechanisms involved in vascular aging. Mitochondrial dysfunction, oxidative stress, mechanical stress, telomere attrition, epigenetic alterations, and deregulated nutrient sensing are relevant mechanisms involved in driving vascular aging. See text for the details. mTOR: mammalian target of rapamycin; SIRT1: sirtuin 1; AMPK: AMP-activated protein kinase; ATP: adenosine triphosphate; ROS: reactive oxygen species; NO: nitric oxide; TGFβ: transforming growth factor β; VSMC: vascular smooth muscle cells.

These age-related changes in ECM affect ECs and VSMCs, because these cells are particularly sensitive to changes in mechanical forces. Aging-dependent stiffening in the endothelial basal membrane increases endothelial permeability and infiltration of inflammatory cells in the aortic wall, predisposing this to atherosclerosis [82]. Similarly, VSMCs rapidly respond to mechanical forces by activating some gene programs and suppressing others, resulting in the loss of contractile properties and the acquisition of a more proliferative and pro-inflammatory phenotype. Large artery compliance is also greatly affected by the lack of turnover in VSMC non-muscle actin polymerization at focal adhesions [69], resulting in the misalignment of VSMC contractile apparatus with the ECM and impaired aortic contractile function. Lastly, in the settings of oxidative stress, which is enhanced with endothelial dysfunction in the aged vasculature, subsets of dendritic cells have been shown to accumulate lipid peroxidation adducts (isoketals), which stimulate T-cell infiltration in the aortic wall, in turn promoting collagen production from adventitial fibroblasts. These findings suggest that targeted anti-inflammatory therapies could be useful to mitigate arterial stiffness [83]. Similarly, the anti-aging molecules with anti-inflammatory and antioxidant effects, such as sirtuin-1 and klotho, have also proven beneficial against arterial stiffening [84,85,86] (Figure 3). 

Animal models, mainly aging, obese, or hypertensive mice, have been essential in identifying molecular mechanisms described above, many of which have been validated in non-human primates. Therefore, despite their limitations, such as the difficulty of mimicking menopause in rodents, which may preclude the accurate study of vascular aging in women, these experimental models remain essential (i) to validate trait loci associated with cardiovascular risks from genome-wide association studies, (ii) to elucidate cause–effect relationships between identified targets and vascular stiffness, (iii) to identify biomarkers to improve the early detection of individuals with high cardiovascular risk, and, most importantly, to develop targeted therapies against vascular aging.

## 5. Hints for a Healthy Vasculature

Only 1% of individuals over 70 years of age maintain normal vascular function [80], highlighting an unmet need to develop specific and personalized interventions for the prevention of vascular aging and associated CVD risk. Healthy aging is the result of many interconnected determinants, from genetics to diet, from exercise to social support [87]. While some risk factors, such as chronological age, sex, ethnicity, and genetics, are non-modifiable, others can be modified to promote healthy aging. This requires that politicians and public administrators foster programs to promote income equality, affordable housing, access to healthcare services, as well as activities that promote social connections and engagement for older adults. Other risk factors can be shaped by focusing on lifestyle interventions for the promotion of healthy vascular aging [88]. 

Although some controversies remain, moderate regular aerobic exercise maintains vascular health and reduces cfPWV in adults due to decreased inflammation and oxidative stress and increase in NO bioavailability [89,90]. Aerobic exercise has also been shown to reduce collagen deposition as well as the formation of advanced glycation end products in the arterial wall [89,90,91,92]. Longitudinal and interventional studies indicate that increased physical activity early in life has beneficial effects in preventing or retarding vascular aging [93]. 

Caloric restriction and reduction of sodium intake have also been shown to markedly decrease cfPWV, through the reduction of ROS formation in the vascular wall and the increase of NO [94,95]. This effect of caloric restriction is independent of blood pressure changes [96]. Furthermore, limiting the intake fructose-based sweeteners, which has substantially increased over the past three decades, could yield favorable outcomes. Notably, an elevated fructose regimen triggers xanthine oxidase-mediated oxidative stress, thereby compromising endothelial function and promoting arterial stiffening [97]. Since fruit and vegetables are rich in vasculo-protective phytochemicals and minerals, increasing their intake is particularly recommended [98], thus paving the way for the use of nutraceuticals to restore and preserve vascular health. Diet plays a significant role in shaping gut microbiota, which is a pivotal modulator of human pathophysiological processes [99]. Dysbiosis has been implicated in the process of vascular ageing and a recent systematic review has documented an association between the composition of gut microbiota and arterial stiffness [100].

Accumulating evidence from experimental studies highlights several potential molecular targets that are dysregulated in aging including mTOR, sirtuins, and AMPK. As mentioned above, these pathways could serve as targets for specific pharmacological interventions [77,78,79].

## 6. Conclusions

Because vascular aging predicts cardiovascular events and mortality, it is necessary to implement early diagnosis to detect asymptomatic individuals at high risk of developing CVD. More studies are warranted to identify the best strategy to quantify vascular aging. In addition to sensitizing policymakers to the importance of social determinant in CVD, it is imperative to identify proper and, if possible, personalized nutritional and pharmacological strategies as well as tailored physical activity programs to prevent, delay, and eventually revert the disease.

## Data Availability

Not applicable.
